# Management of a Patient With Ankylosing Spondylitis and Multiple Comorbidities Who Underwent a Percutaneous Nephrolithotomy Using an Innovative Positioning Strategy

**DOI:** 10.7759/cureus.51455

**Published:** 2024-01-01

**Authors:** Ranjay Mahaseth, Mridul Dhar, Revanth B Challa, Vikas K Panwar, Ravi Chaudhary

**Affiliations:** 1 Anaesthesiology, All India Institute of Medical Sciences, Rishikesh, IND; 2 Anaesthesiology, All India Institute of Medical Sciences, Nagpur, IND; 3 Urology, All India Institute of Medical Sciences, Rishikesh, IND

**Keywords:** pulmonary emphysema, renal calculi, percutaneous nephrolithotomy, bronchoscopy, ankylosing spondylitis

## Abstract

Ankylosing spondylitis (AS) is a chronic inflammatory disorder leading to bony ankylosis, ossification, and fibrosis of the spine. Airway management is difficult in these patients due to restricted head and neck movement as well as a stiff body posture. This also poses challenges in lying down supine as well as surgical positioning. We report a case of a patient with AS and multiple co-morbidities who underwent a percutaneous nephrolithotomy for renal calculi, and the customisations made in anaesthetic and surgical techniques to safely perform the procedure.

## Introduction

Ankylosing spondylitis (AS) is an inflammatory disorder affecting both the axial skeleton and peripheral joints of the body, leading to difficulty in both regional and general anaesthesia [1]. Airway management is challenging in these patients as they have a fixed neutral head position. Percutaneous nephrolithotomy (PCNL) is usually done in the lithotomy and prone positions, and such a case becomes difficult and challenging for both anaesthetists as well as surgeons [2]. We report the anaesthetic management of a case of a 57-year-old male with bilateral renal stone disease, hypertension, chronic kidney disease (CKD), chronic obstructive pulmonary disease (COPD) with large emphysematous bullae, and thoracic kyphosis with fixed cervical joints due to AS who underwent left PCNL under general anaesthesia using an innovative surgical position.

## Case presentation

**Figure 1:**
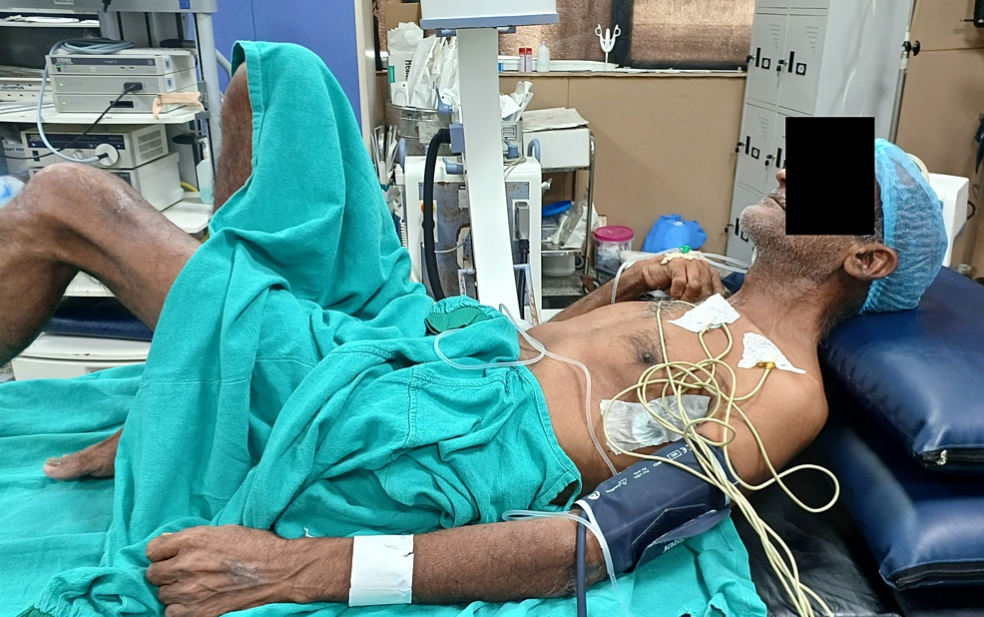
Patient position at rest

A 57-year-old male diagnosed with AS for the past 10 years was planned for PCNL surgery for renal stones. The patient had severe thoracic kyphosis and an immobile cervical spine. Computed tomography (CT) scans showed reduced atlanto-occipital joint space. He also gave a history of a hip injury following which he developed a fixed flexion deformity of the right hip (Figure 1). He had a history of hypertension and CKD (on intermittent haemodialysis) for the past two years along with COPD for the past three years on metered-dose inhalers (levosalbutamol and ipratropium), with shortness of breath on exertion (Modified Medical Research Council grade II). On CT scan, there were thick-walled fibro-cavitary changes. Multiple emphysematous bullae were noted in both lungs, the largest measuring 4.3 x 2.2 cm. Spirometry to assess pulmonary function could not be performed as the patient was unable to forcefully exhale. Breath holding time was 12 seconds. His metabolic equivalent of task score was less than four. On airway examination, his mouth opening was more than 3 cm, but he had a Modified Mallampati grade of III. A difficult airway was anticipated in view of the lack of extension of the atlanto-occipital joint and fixed flexion deformity of the neck and spine overall.

Preoperatively, the patient was optimised with bronchodilation (nebulisation with levosalbutamol-ipratropium respules until the morning of surgery), lung physiotherapy, and counselled about awake flexible bronchoscopy-guided nasotracheal intubation. Informed written consent was taken. The risks were explained for perioperative respiratory complications including an intraoperative high risk of bullae rupture and pneumothorax as well as bony fractures. The neuraxial block was ruled out as it would require a high sensory level, and the patient was unable to guarantee the ability to regulate breathing during the percutaneous approach to the kidney. The patient was also unable to either sit up or lie on the side for the neuraxial block.

On the day of surgery, nil per oral orders were confirmed. After obtaining adequate IV access and preparation of the airway for awake intubation, the patient was shifted to the operation theatre (OT). The airway was secured using a flexible bronchoscope with a flexo-metallic tube of 7.5 mm. A standard awake nasotracheal intubation was done with airway blocks (superior laryngeal nerve and trans-tracheal) and topicalisation of the airway using 4% lignocaine via nebulised route, supplemented with spray as you go via the bronchoscope. The patient was induced with injections of fentanyl 50 mcg, propofol 100 mg, and atracurium 30 mg. Ventilation was maintained with a fraction of inspired oxygen of 0.4, a low tidal volume of 240 mL, and a respiratory rate of 15/min to maintain a minimum minute ventilation to achieve end-tidal carbon dioxide (ETCO2) between 35 and 40 mmHg, while avoiding an increase in airway pressure. The observed peak airway pressure and the plateau pressure were both around 14 cm H_2_O. A low positive end-expiratory pressure of 3 cm H_2_O was applied while monitoring the flow volume curves.

**Figure 2:**
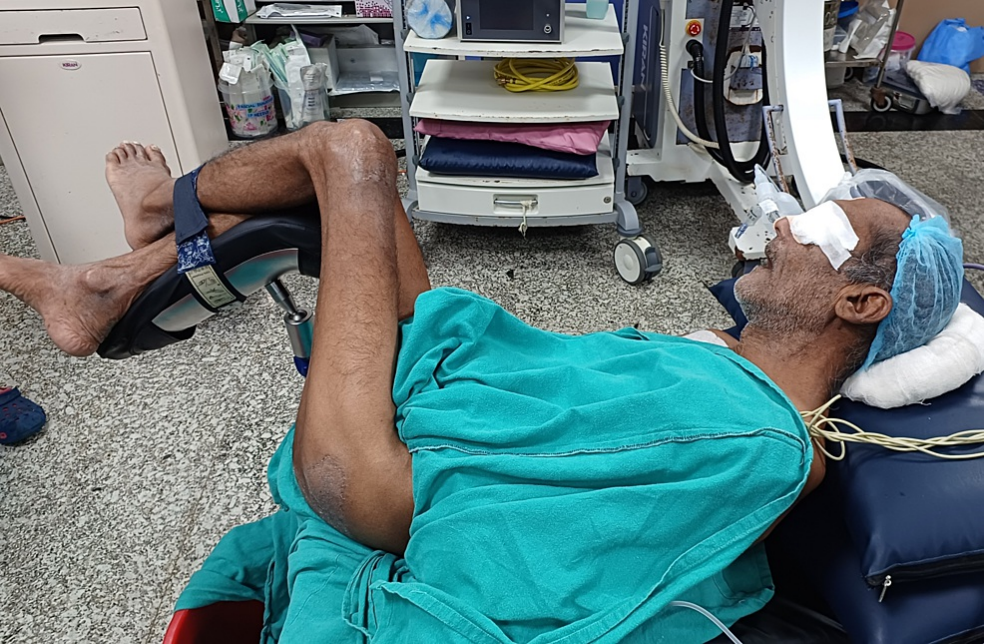
Patient in a modified lithotomy position

Because of the deformed shape of the spine as well as the pelvis, the patient could not lie down supine, and it was virtually impossible to make him prone for the surgery. A conventional lithotomy was also not possible due to a fixed pelvis. After discussion with the surgical team, it was decided that the patient be positioned in a modified lithotomy position with both legs in one stirrup on the right side to minimise manipulation of fixed joints for the initial ureteroscopy (Figure 2). Along with this, pillows were put under the head to support the cervical joint. As this position was as close to his natural resting position while lying down, a supine PCNL was performed in the same position with a slight elevation of the loin with a bolster. The vitals were stable throughout the procedure. Recovery and extubation were uneventful, with no signs suggestive of pneumothorax at the end of the procedure, which was confirmed by C-arm fluoroscopy in OT, and a bedside X-ray later in the ward (which also ruled out any new bony injury).

## Discussion

AS is known to be challenging to anaesthetists as it is an anticipated difficult airway due to restricted neck and spine mobility. There is an increased chance of iatrogenic fractures during intubation, with hyperextension being the most common mechanism of injury and can lead to severe cervical injury even with the use of a video laryngoscope [3]. Therefore, patients with AS with cervical spine fusion should be intubated with awake flexible bronchoscopy as far as possible.

Ossification of axial ligaments and sacroiliac joints, diminished intervertebral gaps, and stiffness of the axial skeleton all contribute to the added risk of fractures while positioning [1]. In the current case, an old fracture further contributed to a fixed flexion deformity at the hip joint on the right side, which made all conventional surgical positioning including lithotomy and proning impossible to execute. Here we wish to highlight the teamwork between the surgeon and the anaesthetist to plan a customised innovative lithotomy position with both legs in one stirrup to minimise any forceful movement on the hip joint. This surgical position was most suitable for this patient considering his natural resting position. As for conventional lithotomy, the vulnerable pressure points should be properly padded along with support to the head and neck. The surgeons also customised their technique to proceed with a supine PCNL under fluoroscopy guidance. Although prone is the surgically safer and conventional option for performing PCNL, the supine technique is also being researched in view of its more stable haemodynamics, better access to the patient, and avoidance of complications of proning [4].

In the current case, in addition to AS, the patient had multiple comorbidities which included CKD, COPD with bullae, and hypertension. Appropriate anaesthetic techniques and modifications in drug doses and drug choices were done along with a lung protective ventilation strategy. When administering anaesthesia to patients with large bullae, precautions to be taken include periodic chest auscultation, vigilantly monitoring ventilator parameters, and allowing permissive hypercapnia if required [5].

## Conclusions

Patients with multiple systemic comorbidities pose a multitude of anaesthetic challenges, which need to be optimised systematically, peri-operatively. Patients with AS require special care and sensitisation among all team members while positioning to prevent iatrogenic injuries. Such patients may require specific customisations and innovations in anaesthetic and sometimes surgical techniques to obtain the best possible outcomes without compromising on safety. A supine PCNL with a single stirrup lithotomy may be considered in this rare subset of patients with bony deformities or restrictions like in AS.
